# Putative Role of Respiratory Muscle Training to Improve Endurance Performance in Hypoxia: A Review

**DOI:** 10.3389/fphys.2018.01970

**Published:** 2019-01-15

**Authors:** Jesús Álvarez-Herms, Sonia Julià-Sánchez, Francisco Corbi, Adrian Odriozola-Martínez, Martin Burtscher

**Affiliations:** ^1^Department of Cell Biology, Physiology and Immunology, Faculty of Biology, University of Barcelona, Barcelona, Spain; ^2^National Institute of Physical Education of Catalonia (INEFC) – Lleida Centre, University of Lleida, Lleida, Spain; ^3^Department of Genetics, Anthropology and Physiology, University of the Basque Country (UPV), Campus de Bizkaia, Bilbao, Spain; ^4^Department of Sport Science, University Innsbruck, Innsbruck, Austria

**Keywords:** respiratory muscles, physical performance, training, muscle endurance, respiratory exercises, hypoxia, adaptation

## Abstract

Respiratory/inspiratory muscle training (RMT/IMT) has been proposed to improve the endurance performance of athletes in normoxia. In recent years, due to the increased use of hypoxic training method among athletes, the RMT applicability has also been tested as a method to minimize adverse effects since hyperventilation may cause respiratory muscle fatigue during prolonged exercise in hypoxia. We performed a review in order to determine factors potentially affecting the change in endurance performance in hypoxia after RMT in healthy subjects. A comprehensive search was done in the electronic databases MEDLINE and Google Scholar including keywords: “RMT/IMT,” and/or “endurance performance,” and/or “altitude” and/or “hypoxia.” Seven appropriate studies were found until April 2018. Analysis of the studies showed that two RMT methods were used in the protocols: respiratory muscle endurance (RME) (isocapnic hyperpnea: commonly 10–30′, 3–5 d/week) in three of the seven studies, and respiratory muscle strength (RMS) (Powerbreathe device: commonly 2 × 30 reps at 50% MIP (maximal inspiratory pressure), 5–7 d/week) in the remaining four studies. The duration of the protocols ranged from 4 to 8 weeks, and it was found in synthesis that during exercise in hypoxia, RMT promoted (1) reduced respiratory muscle fatigue, (2) delayed respiratory muscle metaboreflex activation, (3) better maintenance of SaO_2_ and blood flow to locomotor muscles. In general, no increases of maximal oxygen uptake (VO_2max_) were described. Ventilatory function improvements (maximal inspiratory pressure) achieved by using RMT fostered the capacity to adapt to hypoxia and minimized the impact of respiratory stress during the acclimatization stage in comparison with placebo/sham. In conclusion, RMT was found to elicit general positive effects mainly on respiratory efficiency and breathing patterns, lower dyspneic perceptions and improved physical performance in conditions of hypoxia. Thus, this method is recommended to be used as a pre-exposure tool for strengthening respiratory muscles and minimizing the adverse effects caused by hypoxia related hyperventilation. Future studies will assess these effects in elite athletes.

## Introduction

Early studies on the influence of specific respiratory muscle training (RMT) upon exercise in healthy adults in normoxia provide convincing evidence supporting the ergogenic effect on endurance performance (Markov et al., 2001; Stuessi et al., 2001; Volianitis et al., 2001; Romer et al., 2002a,b; Johnson et al., 2007). However, there are also some studies being more cautious of beneficial RMT effects (Morgan et al., 1987; Fairbarn et al., 1991; Hanel and Secher, 1991) on physical performance. Despite controversy, the avoidance of respiratory muscle fatigue and its systemic and perceptual repercussions may play a crucial role in the health and physical performance. In addition, RMT effects upon improving blood redistribution to limb locomotor muscles during heavy exercise (McConnell and Romer, 2004) are of utmost importance for endurance physical activities. During recent years it has also been postulated that RMT could be able to reduce premature fatigue of respiratory muscles subjected to maximal demand in moderately/highly trained athletes as well as climbers exposed to hypoxia (Verges et al., 2010). Moreover, preliminary results indicate favorable RMT effects in normoxia on endurance and strength of respiratory muscles, and delayed onset of the respiratory metaboreflex (Dempsey et al., 2006), lactate accumulation (Verges et al., 2010), hipoxemia (Downey et al., 2007), and sympathetic activation (McConnell and Romer, 2004). Ideally, these positive responses would be beneficial as an enhancer procedure of the respiratory muscles prior to exposure or competition in hypoxia or at altitude (Downey et al., 2007; Esposito et al., 2010).

Human lowlanders have a normal and programmed physiological response to hypoxia involving different genetic, cellular and systemic regulation (Dempsey and Morgan, 2015). Initially, the decrease of arterial oxygen partial pressure reflexively activates the response of arterial chemoreceptors inducing an increase in the ventilatory response via neural systems (Schoene, 2001). The deficient oxygenation of the blood is known as “hypoxemia” and may be induced by physical exercise (Romer et al., 2006), exposure to hypoxia (Romer et al., 2006) or several types of diseases (San Martin et al., 2017). As a consequence of hyperventilation, metabolic changes (Katayama et al., 2010) as well as altered neural responses (Kayser, 2003) and/or peripheral fatigue mechanism alterations may occur (Kayser et al., 1994). Each individual exhibits a different level of sensitivity to hypoxia, some responding more pronounced than others, with the latter suffering from higher levels of hypoxemia (Chapman and Emery, 1999) and dyspnea (Amann et al., 2006) during (sub)maximal exercise. Related to pronounced hyperventilation or dyspnea during intense exercise in hypoxia, respiratory muscles may become fatigued and the accumulation of metabolites in these muscles activates phrenic afferents thereby increasing sympathetic vasoconstrictor activity in the working skeletal muscles (Harms, 2007). This response is called respiratory muscle metaboreflex (Di Prampero, 1985). Witt et al. (2007) demonstrated in healthy subjects that RMT resulted in a delayed activation of this reflex during exercise in normoxia associated with reduced cardiovascular responsiveness and improved exercise performance. It seems conceivable that RMT should even more beneficially affect exercise performance in hypoxia.

Compared to normoxia (Witt et al., 2007), RMT might be more important in hypoxia due to the particular challenge to the respiratory muscles. Generally, Di Prampero (1985) and Di Prampero and Ferretti (1990) described four stages in which VO_2max_ limitations can occur in both hypoxia and normoxia in the respiratory process: (1) ventilatory, between the environment and the alveoli; (2) pulmonary, between the alveoli and the blood; (3) circulatory, between arterial blood and muscle capillaries; and (4) peripherally, between muscle capillaries and mitochondria. In conditions of hypoxia, the physiological adjustments triggered in stages 1 and 2 cannot be compensated by equivalent changes in stages 3 and 4, as they can in conditions of normoxia. Therefore, although ventilatory function could be relatively well-preserved, this is not able to fully compensate the PaO_2_/SaO_2_ decrease with an existing impairment of the alveolar-capillary oxygen diffusion capacity (Powers et al., 1989; Di Prampero and Ferretti, 1990). Consequently, distinct hyperventilation may occur due to the activation of chemoreceptors (Bernardi et al., 2006). Hyperventilation in hypoxic environment may seriously provoke inspiratory muscle fatigue because muscles work at a shorter than optimal length and at a faster shortening velocity (Dempsey et al., 2008). This effect seems to be due to the lack of adaptation because highlanders for example, have blunted (rather than enhanced) hypoxic chemosensitivity at both rest (Lahiri et al., 1970) and during exercise (Dempsey et al., 1972). These people show only minimal hyperventilation during exercise, but they preserve their PaO_2_ and SaO_2_ at about the same level as observed in considerably hyperventilating lowlanders (Dempsey et al., 1972). In addition, women are more likely to suffer from a limitation in expiratory flow during exercise likely due to their smaller lung volumes and narrower respiratory tract diameters for a given lung volume (Martin et al., 1987). However, in three studies which included women (see Supplementary Table 1) no difference for the respiratory muscle function and performance was reported. Age may also impact on this process because aging leads to a loss in tissue elasticity, thereby resulting in a reduction in the maximal flow volume loop and higher ventilation proportions in the dead space at rest and during exercise (Dempsey et al., 2008). All studies included in this study involved subjects aged below 50 years.

At maximal exercise (above 85% of VO_2max_) (Harms et al., 1997) and during exercise in hypoxia (Downey et al., 2007), activation of the respiratory muscle metaboreflex causes reflex vasoconstriction of the locomotor muscles (Witt et al., 2007; Romer and Polkey, 2008). This is considered as an adaptive mechanism to safeguard pulmonary function and respiratory muscle perfusion in conditions of maximal physiological demand, finally ensuring appropriate oxygenation of the brain and heart (Seals, 2001). During maximal exercise and even submaximal exercise in hypoxia, RMT has been shown to effectively attenuate this reflex (Illi et al., 2012) and to increase blood flow to the locomotor muscles (McConnell and Romer, 2004). This adaptive response helps to lower the perception of exertion in conditions of dyspnea (Romer et al., 2002b) and improves respiratory muscle efficiency (Salazar-Martínez et al., 2017).

### Why Could the Use of RMT Be Useful in Athletes and Climbers Exposed to High Altitude/Hypoxia?

Nowadays, the popularity of hypoxic exposure/training methods has grown exponentially among endurance athletes and climbers who aim to improve physical endurance performance (Álvarez-Herms et al., 2015) and intend to induce the acclimatization process (Ricart et al., 2000; Levine and Stray-Gundersen, 2001). Despite some benefits of hypoxic methods on endurance performance (Bailey et al., 1998; Millet et al., 2010), the individual response to these conditions (Mazzeo, 2008) requires caution when performing such interventions. The hypoxic environment *per se* promotes higher psychophysiological stress in comparison with normoxia, thereby increasing the risks of maladaptive responses in athletes or even that of altitude sickness in climbers (Richalet et al., 2012; Dempsey and Morgan, 2015). These responses are somewhat different between the type of hypoxic exposure [hypobaric (HH) or normobaric (NH)] (Millet et al., 2010). It seems that HH induces an even higher level of stress and adaptation at different physiologic levels: ventilation (Savourey et al., 2003), fluid balance (Loeppky et al., 2005), metabolism (Kayser, 2009), and performance (Bonetti and Hopkins, 2009).

When lowlanders are exposed to either hypoxic condition, the key—the initial/decisive—mechanisms inducing adaptive physiological changes are hyperventilation and hemoconcentration (Bernardi et al., 2001b; Xing et al., 2008), challenging the respiratory and cardiovascular systems. The excess of hyperventilation means a markedly increased work of breathing (Flenley et al., 1979), as well as increased susceptibility to expiratory flow limitation, leading to hyperinflation and severe dyspneic sensations (Bernardi et al., 2006). The energy cost of breathing in hypoxia (Babcock et al., 1995) increases exponentially in comparison to normoxia, and amounts to 15–30% of total maximal oxygen uptake (Bassett and Howley, 2000) and 14–16% of total cardiac output (Downey et al., 2007). Under these circumstances, the same activity performed in hypoxia increases the respiratory muscle stress and promotes faster fatigue in comparison normoxia. Consequently, respiratory muscle fatigue in hypoxia represents a limiting factor of physical performance (Vogiatzis et al., 2007; Verges et al., 2010) in athletes and climbers.

In order to minimize impairment of exercise performance during hypoxia exposure, the process of acclimatization is required to promote physiological adjustments of the respiratory, cardiovascular, hematologic, metabolic, and neural systems (Green et al., 1992; Lyons et al., 1995; Nummela and Rusko, 2000; Ricart et al., 2000; Levine and Stray-Gundersen, 2001; Dempsey et al., 2014). Under these conditions, subjects are commonly advised to follow various recommendations: (1) to rest adequately, (2) to optimize nutrition and hydration, (3) iron intake and, (4) to perform only moderate physical activity. Recently, the inclusion of RMT has been proposed as a preparatory method to enhance the respiratory muscle efficiency 4–6 weeks before athletes and climbers are exposed to hypoxia/altitude (Downey et al., 2007; Esposito et al., 2010; Lomax, 2010; Helfer et al., 2016; Lomax et al., 2017; Salazar-Martínez et al., 2017).

The aim of this review is to provide, (1) a brief description of the respiratory function during hypoxic exposure, (2) a brief description of the techniques of RMT, (3) an overview of the published literature pertaining to the effect of RMT upon exercise performance in hypoxia, (4) an insight into putative mechanisms underlying the ergogenic effects, and (5) suggestion for future investigations.

## Method

A literature search was done in the electronic databases PubMed and Google Scholar using the keywords: “Respiratory/Inspiratory muscle training” AND “endurance performance” AND “hypoxia” OR “altitude”. Articles were selected according to the following criteria: published at any time before 18 April 2018; interventions in healthy active or sedentary people with hypoxic exposure; protocols including a control group (sham or placebo) or not; studies assessing exercise performance (pre- vs. post-intervention); and protocols based on specific respiratory muscle strengthening exercises. The following items were excluded: literature reviews; conference presentations; short communications and papers; references of book chapters or whole books; and articles not written in English. Due to the small number of studies published in the field, some criteria such as age, gender, residence altitude, or weight were not considered. All the data and methodological variables of the selected articles were initially recorded and analyzed by the first author and further discussed with co-authors.

## Results

### Effects of RMT on Exercise Performance in Hypoxia

All of the cross-sectional studies discussed here (see Supplementary Table 1) demonstrated that RMT could bean useful tool for improving ventilatory efficiency and delaying the onset of premature fatigue during exercise in conditions of hypoxia (Downey et al., 2007; Esposito et al., 2010; Lomax, 2010; Helfer et al., 2016; Lomax et al., 2017; Salazar-Martínez et al., 2017). Only Esposito et al. (2010) were somewhat cautious with the benefits of RMT for physical performance in hypoxia. They showed that after RMT, ventilatory parameters were improved (>12% expired volume, >13% alveolar ventilation, >75% MIP, increased pulmonary function, static and dynamic volumes, and alveolar-arterial gradient) during hypoxia. However, submaximal cycling performance was not significantly enhanced. Study findings regarding the effects of RMT upon endurance performance in hypoxia remain scarce in comparison with the large number of investigations performed in normoxic conditions (Illi et al., 2012). In normoxia, most of the studies suggested to be useful for improvement of respiratory muscle efficiency (static and dynamic) and endurance performance (Illi et al., 2012).

Characteristics like the type of sport, physical fitness of subjects, type and duration of exercise (constant or intermittent exercise) and gender/age may be relevant for the comparability of published results. Supplementary Table 1 summarizes general results of studies on RMT and hypoxia.

### Types of RMT Protocols

Two RMT protocols have been described in the studies included in this review: (1) Respiratory muscle strength (RMS) training (resistive load on inspiration); and (2) Respiratory muscle endurance (RME) training (resistive load on both expiration and inspiration at the same time) (ventilatory isocapnic hyperpnea). RMS includes high-force and low-velocity contractions, generating maximal muscle pressure capacity on inspiration and expiration against resistance (Leith and Bradley, 1976). In contrast, RME consists of high-velocity low-resistance contractions, mainly stimulating the expiratory muscles (Leith and Bradley, 1976). It has not been determined whether one method is more valid than the other, because their applicability in both hypoxia and normoxia depends on several aspects (Illi et al., 2012): (1) sport type (the athlete's biomechanical position during action) and respiratory muscle demand in the active position; (2) exertion time; (3) exertion modality (constant or gradual/intermittent); and (4) exercise intensity. The findings from studies in normoxia have shown that RMT with isocapnic hyperpnea (RME) is more useful for preventing respiratory muscle fatigue (Johnson et al., 1993), while the use of devices to improve RMS (Powerbreathe) is more useful for intermittent exercises (Romer et al., 2002a). Of the studies included in the present work, three focused on RME and four on RMS. In both cases, it was found that specific aspects of their training stimulus had improved: RME, respiratory muscle endurance and RMS, respiratory muscle strength—maximal inspiratory power. The protocols usually described are similar in all studies included in this review: for respiratory muscle endurance (RME; isocapnic hyperpnea: 10–30′ during 3–5 days per week) and respiratory muscle strength (RMS; always employing Powerbreathe devices: 2 × 30 repetitions per day for 5–7 days per week at 50% of maximal inspiratory pressure). Commonly both protocols were performed during 4 weeks.

### Ventilatory Function During Hypoxia After RMT

In lowlanders exposed to hypoxia, the respiratory mechanical demand rapidly increases during the acclimatization process, especially during exercise intensities above 70% (Fitting, 1991). The rising hypoxic ventilatory response (HVR) helps to perform aerobic work at altitude (West, 2000). The fact that each subject may respond differently to hypoxia is of particular interest because one subject will benefit more from the RMT than another.

All included studies analyzing RMT effects demonstrated some positive change in pulmonary function in hypoxia (see Supplementary Table 1). However, in some cases no direct translation of these changes was shown into exercise improvement. The considerable capacity of the respiratory muscles to adapt to training can be observed by the significant increase in maximal inspiratory power (MIP) after stimuli such as exercise training (Coast et al., 1990), RMT (Illi et al., 2012), and also exposure to hypoxia (Babcock et al., 1995). MIP means inspiratory muscle strength, a useful measure to diagnose pulmonary impairment or improvement (Coast et al., 1990). Studies analyzing the effect of RMT on MIP in hypoxia found positive changes when RMS and RME was the aim of the training protocol (Illi et al., 2012). Such changes may well represent broad improvements in exercise performance and health in sedentary and physically active subjects as well (Volianitis et al., 2001; Johnson et al., 2007; Tong et al., 2008), albeit less so than in highly-trained subjects (Coast et al., 1990). Lomax (2010) and Lomax et al. (2017) showed, compared to the control group, improved MIP (~15%) and minute ventilation (21%) in hypoxia within the RMS training group (using Powerbreathe). However, maximal expiratory power (MEP) did not change in either group. Downey et al. (2007) also demonstrated a 24.5% improvement of MIP with an equivalent increase in RMS (25%). In the same study, six of the seven assessed subjects had improved their respiratory muscle fatigue endurance capacity and time to fatigue. This aspect is important because, after exercise in hypoxia, MIP decreases proportionally to respiratory muscle fatigue (up to 17%) (Downey et al., 2007). In the study by Salazar-Martínez et al. (2017), the authors suggest that improved MIP could positively alter respiratory patterns during exercise in hypoxia without significantly increasing the minute volume/CO_2_ volume gradient curve. These authors reported an increase of ~28% MIP in hypoxia with a RME protocol. In concordance with Esposito et al. (2010) and Salazar-Martínez et al. (2017) also assumed improved alveolar-ventilation and the alveolar-arterial gradient in hypoxia after RME. In concordance, Bernardi et al. (2006) demonstrated higher ventilatory efficiency and “optimized” breathing patterns in Mount Everest and K2 climbers ascending without oxygen compared to those needing oxygen. Lomax et al. (2017) showed lowered minute ventilation, CO_2_ volume and SaO_2_, in those with improved MIP thereby improving ventilatory efficiency during constant exercise in hypoxia (cycling). It is to highlight that such changes in MIP were only observed in the RMT groups but not in the control groups. Conversely, Esposito et al. (2010) reported after a RMS protocol (without control group) improved MIP by around 50%, but there were no changes in exercise performance. The effects of RMT on MIP in normoxia are similar, although values are lower (10–18%) in rowing (Volianitis et al., 2001) and cycling (Romer et al., 2002b). MIP measurement is highly relevant to assess the respiratory efficiency after different RMT protocols; however, there seems not to be a direct relation with better exercise performance neither in normoxia nor hypoxia.

### VO_2max_ and SaO_2_

When ascending to high altitude, the partial pressure of oxygen (PO_2_) falls and hypoxemia develops within minutes of arrival. To limit the drop in the arterial oxygen content, cardiac output is elevated through sympathetic activation and alveolar ventilation is increased due to stimulation of peripheral chemoreceptors (San Martin et al., 2017). Nevertheless, aerobic exercise capacity decreases (Saunders et al., 2009), related to both a decrease in arterial oxygen content and a limitation in maximal cardiac output (Fulco et al., 1998). Poor ventilatory efficiency has to be compensated by hyperventilation to maintain appropriate SaO_2_ levels (Rusko et al., 2004; Burtscher et al., 2006) but sometimes leading to respiratory muscle fatigue. In addition, the decrease in maximal cardiac output at altitude has been explained by the combined effects of decreased blood volume, hypocapnia, increased viscosity of the blood, autonomic nervous system changes, and/or depressed myocardial function (Wagner, 2000). An additional factor might be a limitation in right ventricular flow output secondary to hypoxic pulmonary hypertension. Actually, an improvement in maximal workload and maximal oxygen uptake (VO_2max_) together with a decrease in pulmonary artery pressure (PAP) was reported after the intake of medication as sildenafil or dexamethasone in hypoxic healthy volunteers (Ghofrani et al., 2004). However, improved exercise capacity in these studies could not unequivocally be ascribed to associated inhibition of hypoxic pulmonary vasoconstriction, because of additional effects including a variable improvement in arterial oxygen content (Richalet et al., 2005). Alveolar hypoxia promotes the adaptive vasomotor response defined as hypoxic pulmonary vasocontriction (HPV), which redistributes blood to optimally ventilated lung segments by an active process of vasocontriction, particularly involving the small, muscular “resistance” pulmonary arteries (Moudgil et al., 2005). HPV may be disadvantageous in global hypoxia due to a substantial increase in pulmonary vascular resistance and pulmonary arterial pressure (Bärtsch and Gibbs, 2007). Genetic adaptation may favor highlanders to have lower pulmonary arterial pressure in hypoxia compared with lowlanders (Stuber et al., 2008). However, HPV is significantly improved by hyperventilation (Bindslev et al., 1985). Taking into account that hyperventilation in hypoxia promote respiratory muscle fatigue and premature decrease in exercise performance, RMT could be an useful tool to minimize the effects of HPV in hypoxia during acclimatization phases and or during maximal physical effort. At the same time, RMT may also favor a more efficient breathing pattern, thereby improving the level of alveolar ventilation and maintaining SaO_2_ values (Bernardi et al., 2006). RMT has been demonstrated to be effective in reducing breathing rate (and elevating tidal volume) at sea level (Bernardi et al., 2014) and at altitude as well (Keyl et al., 2003). Indirectly, slower breathing reduces the HVR (Bernardi et al., 2001a) and diminishes the heart rate response and systemic blood pressure at altitude. Lower ventilatory needs and sympathetic activation could help to maintain exercise capacity at extreme altitudes (Somers et al., 1991). Long term adaptive breathing patterns are found in professional endurance athletes which are highly efficient, since tidal volume don't seem to reach a plateau at high exercise intensities (Luciá et al., 1999). In contrast, normal healthy humans increase minute ventilation by higher breathing frequencies with tidal volume showing a plateau or even a slight decrease (Martin and Weil, 1979; Clark et al., 1983). Witt et al. (2007) suggested that respiratory muscles do not limit maximal oxygen uptake when they are well trained, but would promote a more efficient gas exchange (better ventilation-perfusion and alveolar-capillary exchange) (Salazar-Martínez et al., 2017). Consequently, delayed sensation of dyspnea and an attenuation of the respiratory muscle metaboreflex will occur. Salazar-Martínez et al. (2017) and Esposito et al. (2010) specifically suggested improve ventilation-perfusion and alveolar-arterial gradient in hypoxia after RMT (RMS). RMT has not be shown to be directly effective in improving parameters such as stroke volume, cardiac output or VO_2max_ in either normoxia (Edwards et al., 2008) or hypoxia (Downey et al., 2007; Esposito et al., 2010; Salazar-Martínez et al., 2017). However, Keramidas et al. (2011) (combining RMT with aerobic exercise) and Lomax et al. (2017) showed slight improvements in VO_2max_ during exercise in hypoxia. Interestingly, by strengthening the respiratory muscles with RMT, the oxygen cost in those muscles seem proportionally to decrease, thereby enabling greater oxygen availability for the locomotor muscles and improving motor recruitment via the central nervous system (Edwards and Walker, 2009). Salazar-Martínez et al. (2017) noted that subjects who showed a lower O_2_ cost for the same increase in minute ventilation were those who performed better in cycling time trials. In contrast, Esposito et al. (2010) reported, during maximal exercise in hypoxia, decreased post-RMT VO_2max_ (−23%) and maximal power (Watts) (−20%) compared to normoxia. Although two studies found improved VO_2max_ in hypoxia after RMT, it remains uncertain whether RMT does really provide direct benefits on aerobic capacity and maximal performance in hypoxia/at altitude.

Of the seven studies analyzed (see Supplementary Table 1), only one (Esposito et al., 2010) did not report beneficial post-RMT changes in SaO_2_. Moreover, these improvements were demonstrated after RMT during exercise at moderate (3,000 m) (Kleinsasser et al., 2004) as well as high altitudes (>5,000 m) (Lomax, 2010). The mechanisms promoting a post-RMT rise of SaO_2_ in hypoxia are not fully understood. However, different hypotheses have been proposed, such as alterations in pulmonary capillary red blood cell transit time and/or improvement in the ventilation-perfusion relationship (Bender et al., 1989; Lomax et al., 2017). In this regard, Lomax et al. (2017) reported that post-RMT ventilatory efficiency improved (SaO_2_/minute volume ratio) during exercise in hypoxia, indicating less ventilatory needs to maintain a certain SaO_2_ level. Downey et al. found a post-RMT increase in pulmonary diffusion capacity (23%) as the cause for improved SaO_2_ (Downey et al., 2007). These changes globally would modify the inputs perceived by the peripheral chemoreceptors and in turn, proportionally reduce minute ventilation (Downey et al., 2007) thereby triggering a lower perception of dyspnea and sympathetic activation (Amann et al., 2006). This is an important aspect, since in conditions of hypoxic or maximal exercise the inspiratory muscles work at around 90% of their available capacity to generate pressure (Aaron et al., 1992). In athletes and mountaineers, the potential to perceive respiratory discomfort and fatigue to a lesser extent represents an important element in order to better tolerate the environmental conditions and maintain exercise performance (Noakes, 2000; Noakes et al., 2005).

### Metabolic Acidosis and Metaboreflex

The increased anaerobic metabolism induced by exercise in hypoxia consequently increase the level of circulating metabolites, e.g., lactate and hydrogen ions, in active muscles (including the respiratory muscles). In response to this metabolic acidosis, the fall of arterial pH is constrained by the magnitude of the compensatory hyperventilation (Rausch et al., 1991). Oren et al. (1981) demonstrated that enhanced ventilatory drive resulting from metabolic acidosis also accelerated the ventilatory kinetics during subthreshold square-wave exercise. It has been shown that RMT upregulates monocarboxylate transporters 1 (MCT1) and 4 (MCT4) (McConnell and Sharpe, 2005) and the removal of lactate and hydrogen ions from respiratory muscle fibers (McConnell and Sharpe, 2005; Brown et al., 2012). In normoxia, post-RMT lactate levels are reduced during moderate-to-high-intensity exercise (Illi et al., 2012). Moreover, improved tolerance to acidosis after RMT may contribute to the attenuation of the metaboreflex and reduce premature fatigue occurring during exercise in hypoxia (Witt et al., 2007). Taken together, the metabolic efficiency after RMT may improve the control of blood gases and pH homeostasis during prolonged and intensive exercise in any environmental condition (Lucia et al., 2001).

The connection between muscle contraction and cardiorespiratory responses is evident from the known activation in the discharge frequency of metabosensitive group III/IV, muscle afferents projecting to the cardiorespiratory control centers in the CNS (Amann and Kayser, 2009). This “exercise pressor reflex” (the feedback component) plays, next to central command (the feedforward component) (Waldrop et al., 2011), a key role in the neural control mechanisms determining the proper cardiorespiratory response to exercise. The metaboreflex originating from respiratory muscles attenuate blood flow to the working limb muscles in favor of respiratory muscles (Hansen et al., 2000). Metaboreflex attenuation with the use of RMT is currently a method achieving a broad consensus in the scientific literature. It supports the application of this method to improve respiratory and exercise performance (González-Montesinos et al., 2012). RMT improves clearance capacity and tolerance of lactate and hydrogen-ion levels, especially of the respiratory muscles (McConnell and Sharpe, 2005; Johnson et al., 2007; Brown et al., 2012). In conditions of normoxia, this aspect has been described as a positive one for improving recovery during high-intensity intermittent exercises (Brown et al., 2010) with a constant load and of a time-trial nature (Johnson et al., 2007), increasing the length of time to exhaustion (Gething et al., 2004), regardless of gender (Guenette et al., 2006). In addition, it helps to improve metabolic thresholds and training paces in stable lactate conditions (Brown et al., 2012). In hypoxia, greater respiratory muscle fatigue increases the energy cost of the respiratory process, thereby increasing competition for blood redistribution and decreasing exercise capacity. Thus, strengthening the respiratory muscles directly contributes to a delayed onset of respiratory muscle fatigue by attenuating the onset of reflex vasoconstriction (Harms et al., 1997, 1998; Romer et al., 2006). Debevec and Mekjavic (2012) demonstrated that after four intermittent normobaric exposures (sessions) VE and SpO_2_ (+5%) increased during exercise in hypoxia without affecting high intensity endurance performance. However, the increased exercise ventilation did not result in a significant alteration of the regional cerebral and muscle oxygenation pattern or regional blood volume redistribution from the working leg to the respiratory muscles during hypoxic exercise.

### Perceptive Discomfort of Dyspnea

Loading and unloading of the respiratory muscles during intense exercise have substantial effects on the perception of both respiratory and limb effort (Suzuki et al., 1995; Nicola et al., 2016). The increased central motor command to the locomotor and to the respiratory muscles may be directly perceived via central corollary discharge as an increased sense of muscle effort (Gandevia, 1988). Furthermore, discomfort of limb and respiratory muscles may also arise from the periphery via activation of muscle type IV afferents as muscle metabolites accumulate with the onset of fatigue (Harms et al., 2000). Increasing respiratory muscle work is related to increasing respiratory discomfort (O'Donnell et al., 1999) associated with impaired physical performance (Harms et al., 2000). Due to higher demand of blood flow to the respiratory muscles in hypoxia, benefits argued from RMT may be of interest to reduce perceptive dyscomfort. Although nearly all studies indicate in some way the lower effort perception during exercise in hypoxia after RMT, only Downey et al. (2007) presented data on the ratings of effort perception and dyspneic sensations (*p* < 0.05), which were significantly decreased in the RMT group.

### Discussion

This review provides evidence for RMT to be an effective stimulus for improving strength and endurance (with RME or RMS protocols, see Supplementary Table 1) of the respiratory muscles. These adaptive responses contribute to improved ventilatory function/efficiency very likely translating into exercise performance improvements in normoxia and particularly in hypoxia (Illi et al., 2012; Sales et al., 2016). Thus, it seems evident that RMT has the potential to minimize at least some of the limiting factors related to the respiratory system occurring during training/competition at altitude/in hypoxia. Expected benefits may include, (1) delayed onset of premature fatigue, (2) delayed respiratory muscle metaboreflex onset/activation, (3) improved clearance and tolerance to anaerobic metabolite products, (4) decreased perception of dyspnea, (5) increased SaO_2_ values, and (6) more favorable blood redistribution to the locomotor muscles. Both normal trained persons and elite athletes may benefit from RMT. Particularly in some diseases as obesity, the main pathophysiological mechanisms involved in the impairment of the uptake and management of oxygen include a heightened demand for ventilation, increased work related to breathing, respiratory muscle inefficiency, and diminished respiratory compliance (Parameswaran et al., 2006). Causes for these respiratory abnormalities in obesity include a decrease in total respiratory system compliance, (1) decreased chest wall compliance due to the accumulation of fat in and around the ribs, the diaphragm, and the abdomen (Naimark and Cherniack, 1960). As BMI increases (particularly in morbidly obese individuals), there is evidence of a reduction in expiratory flow and a decrease in forced expiratory volume in 1 second and forced vital capacity (Jaoude et al., 2012). In these conditions, RMT could also be an important tool to improve respiratory muscle function and exercise performance.

Of the analyzed studies in this review only Downey et al. (2007) assessed exercise performance on a treadmill whereas the others used cycle ergometry. This aspect is important because the cycling position has been described as highly stressful for the respiratory muscles promoting premature fatigue (Romer et al., 2002b; Johnson et al., 2007; Hellyer et al., 2015). It is also relevant to note that the hypoxic conditions used in these studies (FiO_2_ of 11–16, see Supplementary Table 1) were slightly higher in comparison with the common altitude used for training/exposure of athletes (between 1,800 and 2,800 m). A delay in the onset of premature fatigue in hypoxia is the key point described in post-RMT improvement of exercise performance (Babcock et al., 1995; Gudjonsdottir et al., 2001; Downey et al., 2007). This may be primarily explained by a reduced respiratory muscle metaboreflex after RMT (Romer et al., 2006). During endurance exercise at a constant pace, Helfer et al. (2016) and Keramidas et al. (2011) demonstrated increased exercise time in hypoxia post RMT (44 and 36% at 75 and 80% of VO_2max_) when compared to the control group. The delayed onset of fatigue was partly attributed to the attenuation of reflex vasoconstriction of the locomotor muscles and a decrease in metabolite accumulation (Leddy et al., 2007).

Breathing during exercise in hypoxia (FiO_2_ equal to 0.15) is associated with increased energy costs (20–30%) when compared to normoxia (Babcock et al., 1995), thereby more likely causing respiratory muscle fatigue (Verges et al., 2010). Downey et al. (2007) also confirmed 20–30% higher ventilatory demands in hypoxia compared to normoxia (FiO_2_: 0.14). Hyperventilation is one of the factors contributing to the more pronounced perception of fatigue and dyspnea (Morgan et al., 1987; Fairbarn et al., 1991). Lomax et al. (2017) proposed hyperventilation during exposure to hypoxia as an useful adaptive response to promote improved inspiratory muscle efficiency and cycling performance. Associated with the improved respiratory efficiency after RMT, blood flow to the locomotor muscles seems to be better maintained (Harms et al., 1997; McConnell and Romer, 2004), thereby reducing the sensation of peripheral fatigue (Suzuki et al., 1995; Volianitis et al., 2001; Romer et al., 2002b; Edwards et al., 2008). Although perceived exertion is extremely important for exercise performance in hypoxia (Álvarez-Herms et al., 2016), it has not been received much attention. The perception of exertion is connected with the afferent feedback from lung receptors to respiratory control centers in the brain, which may influence performance capacity depending on the positive or negative interpretation (Noakes, 2004). RMT helps to integrate this sensory feedback and to expand the ranges of tolerance to exertion (St Clair Gibson et al., 2006). In addition, RME has been reported to provide benefits by improving processing speed and working memory during exercise at altitude vs. placebo/control (Quackenbush et al., 2016). Summarizing these findings, RMT has the potential to improve exercise performance in hypoxia and should therefore be considered as part of the preparation for training or competing at altitude. Based on the relevant studies available, RMT protocols should be based on cycles of five sessions a week for 4–8 weeks of RMS (30–40 reps/d) or RME (20–30′ at 50% MIP).

## Limitations of the Study

It has to be pointed out, that studies currently published about RMT and hypoxia were a small number, subjects and/or conditions are dissimilar and did not assess elite athletes. In the included studies only the one by Salazar-Martínez et al. (2017) considered amateur cyclists. The remaining investigations included sedentary or physically active subjects with a large capacity to improve their exercise and ventilatory function. In this respect, and unlike to studies conducted in normoxia [cycling (Sonetti et al., 2001; Romer et al., 2002b; Holm et al., 2004) and rowing (Volianitis et al., 2001; Wells et al., 2005)], we encountered limitations in extrapolating the results to elite athletes or high-altitude climbers. Methods used to assess post-RMT changes (respiratory muscle strength vs. endurance) in respiratory and exercise performance were not homogeneous and consequently, findings varied markedly. Thus, future studies should assess individual RMT effects in well-trained athletes and professional climbers using standardized RMT protocols and performance tests. No study was aimed to compare the hypoxic type of exposure (HH or NH) and consequences of respiratory muscle function after RMT. For instance, Savourey et al. (2003) found that HH leads to more pronounced hypoxemia, hypocapnia, blood alkalosis and a lower SaO_2_ in comparison to NH. Despite these differences in adaptive responses, the possibility that RMT would more effectively help to maintain exercise performance in HH than NH has not been evaluated.

## Conclusions

In conclusion, RMT potentially represents an appropriate method to improve respiratory and exercise performance in hypoxia/altitude. RMT effects may include (1) reduced respiratory muscle fatigue, (2) delayed respiratory muscle metaboreflex activation, (3) better maintenance of SaO_2_ and blood flow to locomotor muscles. However, RMT effects on exercise performance at altitude/in hypoxia in elite athletes have to be established, especially considering individual needs depending on baseline characteristics, type of sport and hypoxic environmental conditions (HH or NH).

## Author Contributions

All authors listed have made a substantial, direct and intellectual contribution to the work, and approved it for publication.

### Conflict of Interest Statement

The authors declare that the research was conducted in the absence of any commercial or financial relationships that could be construed as a potential conflict of interest.

## Supplementary Material

Supplementary Table 1 Summary of the studies included in the revision: Respiratory muscle training and performance in hypoxia. RMT, respiratory muscle training; VO_2max_, maximal oxygen uptake; resp/d, respirations per day; MIP, maximal inspiratory pressure; MVV, maximal volume ventilation; TT, time trial; N/A, not available; FiO_2_, inspired fraction of oxygen; n, number of participants; SpO_2_, peripheral blood oxygen saturation; VE, expired minute volume; RER, respiratory exchange ratio; RAW, total airway resistences; VT, tidal volume; PPO, peak power output; TT, time trial; VE_max_, maximal minute ventilation; WTT_mean_, mean of watts during time trial; W/kg, watts per kilogram; FVC, forced vital capacity; FEV1, forced expired volume in the first second; PEF, peak expiratory flow; VC, vital capacity; ERV, expiratory reserve volume; RV, residual volume; HR, heart rate; cmH2O, centimeters of water; VCO_2_, carbon dioxide volume; HR_max_, maximal heart rate; HH, hypobaric hypoxia; NH, normobaric hypoxia.
