# Supplementary material for: Putative Role of Respiratory Muscle Training to Improve Endurance Performance in Hypoxia: A Review
Source: Front Physiol. 2019 Jan 15;9:1970. doi: 10.3389/fphys.2018.01970 (PMC6341067; doi:10.3389/fphys.2018.01970)
Supplement: Supplementary Table 1 — Summary of the studies included in the revision: Respiratory muscle training and performance in hypoxia. RMT, respiratory muscle training; VO2max, maximal oxygen uptake; resp/d, respirations per day; MIP, maximal inspiratory pressure; MVV, maximal volume ventilation; TT, time trial; N/A, not available; FiO2, inspired fraction of oxygen; n, number of participants; SpO2, peripheral blood oxygen saturation; VE, expired minute volume; RER, respiratory exchange ratio; RAW, total airway resistences; VT, tidal volume; PPO, peak power output; TT, time trial; VEmax, maximal minute ventilation; WTTmean, mean of watts during time trial; W/kg, watts per kilogram; FVC, forced vital capacity; FEV1, forced expired volume in the first second; PEF, peak expiratory flow; VC, vital capacity; ERV, expiratory reserve volume; RV, residual volume; HR, heart rate; cmH2O, centimeters of water; VCO2, carbon dioxide volume; HRmax, maximal heart rate; HH, hypobaric hypoxia; NH, normobaric hypoxia. [file Table_1.DOCX]

| Study | Methods | RMT-Effects on physical and physiological performance | RMT-Effects on ventilatory function | RMT-Effects on RPE in hypoxia | Benefits of RMT-Effects on SaO_2_ in hypoxia | Hypoxic conditions | Exercise testing device |
| --- | --- | --- | --- | --- | --- | --- | --- |
| Helfer et al.(Helfer, Quackenbush, Fletcher, &Pendergast, 2016) | 15 physically active, non-competitive subjects.  Isocapnic hyperpnea  RMT group: (n=5) 30’/d, 3d/week. 40% ventilatory workload increment in weeks.  Control group (n=5) 30’/d, 3d/week maximal resistance.  4 weeks. | Post-RMT endurance performance at 3,600 m increased + 44% at 75% VO_2max_ (pre-post p<0.001).  9 of 10 subjects improved time to exhaustion after RMT.  No changes in the control group (pre-post p>0.21).  > tolerance to endurance fatigue. | Pre-RMT V̇E increased 21–27% during the initial 12 min of exercise, after which it decreased 17% at 17.7 min. V̇E at altitude post-RMT increased more (49%) for longer (21 min) and decreased less (11% at 25.4 min).  ↓ Respiratory muscle fatigue; because VE maximal expiratory values were occurred pre at minute 12 and post minute 21.  Control group did not showed significant changes.  HR values did not significant between RMT and control (p=0.53). | N/A | NO | 3000m (one group),  3600m other two groups (HH) | Cycle ergometer incremental test:  From 50w, stages of 2 minutes and increasing 50w each 2 minutes until exhaustion. |
| Lomax et al.(Lomax, 2011) | 14 physically active subjects (12 men, 2 women).  Powerbreathe:  RMT group (n=7) 2x30 resp/d at 50-60% MIP 2/d, 7d/week. Load adjusted weekly.  Sham group (n=7) 30 resp/d at 15% MIP 2/d, 7d/week.  4 weeks. | Incremental test: RMT group increased +18% vs. sham +6%. | MIP: RMT group ↑ 15% vs. sham (no changes in the control group). | NO | YES  (> 6% in 4880-5550m)  NO changes in 1400m. | From 1400-4880-5550 m (HH) | Climbing Mountain |
| Lomax et al.(Lomax, 2017) | 17 physically active men.  Powerbreathe.  RMT group (n=8) 2x30 resp/d at 50% MIP, 7d/week.  Placebo group (n=9) 2x30 resp/d without resistance, 7d/week.  4 weeks. | ↓ physiological demand during moderate exercise in conditions of hypoxia after following an RMT protocol.  RMT did not produce any improvements in normoxia.  Conclusions: effective stimulus for preparing subjects to exercise in hypoxia. | An interaction was observed between RMT group for MIP (p = 0.011).  Sham RMT had no effect on MIP (P = 0.715). MIP was increased by 21 ± 16 cmH^2^O in the RMT group following RMT (p = 0.008).  RMT but not sham was associated with a reduction in VE (p = 0.001) and VCO_2_ (p = 0.042) during hypoxic, but not during normoxic exercise.  SpO_2_ (p = 0.004) and SpO_2_/VE (p = 0.005) were increased following RMT during hypoxic exercise.  Dyspnea fell following both hypoxic and normoxic exercise in response to both RMT and sham (p = 0.001) but no difference was observed in dyspnea between groups.  ↑ minute ventilation (21%) in hypoxia. | YES | YES (3%) | FiO_2_ (0.146) (NH) | Cycle ergometer:  10 minutes fixed intensity at 100 watts power. |
| Keramidas et al.(3) | 18 physically active subjects.  Isocapnic hyperpnea.  Aerobic training 1h/d at 50% maximal aerobic capacity, 5d/week and both group: RMT group (n=9), 30’ post aerobic training.  Control group (n=9) no RMT,  4 weeks | VO_2max_ increased in both groups after intervention in normoxic exercise. However in hypoxia, only RMT group increased VO_2max_ during exercise in hypoxia (15.2% vs. -6.5% (control group).  The RMT group increased exercise time at a constant load (80%), 36.7% more than the control group in hypoxia.  Constant-load performance was maintained for 10d post training in the RMT group (34.6%) vs. control.  Hematology did not changed significantly after intervention in any group.  The RMT group had a ↓ HR_max_ (approximately 7  beats min-1) in VO_2max_ in normoxia during Post and After tests, whereas the control group did not. | During the VO_2max_ test in normoxia, the RMT group exhibited a significantly (P<0.05) higher VE_max_ during the Mid and Post compared to Pre testing period.  The RMT group also had significantly  (p<0.05) higher VE_max_ during the Post training hypoxic tests compared to the Pre training values. | YES  RMT group perceived the submaximal exercise training harder than the  Control group (p<0.05) with  higher values of dyspnea and muscle fatigue perceived. | YES (3%) | FiO_2_ 0.12  (NH) | Cycle ergometer:  80% of VO_2max_ until exhaustion. |
| Downey et al.(4) | 15 physically active subjects, 8 men and 7 women.  RMT group (n=7), 40 maximal inspirations from residual volume 2/d, 5d/week, 50% MIP.  Control group (n=5) at 15% MIP.  4 weeks | No increased time to exhaustion after IMT in hypoxia in any group.  Significant improvements on physiological parameters were only found for RMT when exercise testing was performed in hypoxia: lower inspiratory muscle fatigue (7.5%), (14%) lower cardiac output, 22% improved lung diffusion capacity, VE (25%) and VO_2,_ (8-12%).  Lactate concentrations after submaximal tests were not different comparing groups and conditions.  No differences between genders were observed. | ↑ Diaphragm thickness (8-12%) in the RMT group.  MIP increased after RMT 7.5% vs. sham in hypoxia (p<0.05) without changes in normoxia.  Inspiratory muscle fatigue following exercise was reduced  ±10% (P < 0.05) in RMT after both normoxia and Hypoxia. | YES  RPE and dyspnea were lower in the RMT after training in normoxia and hypoxia.  Control group did not showed significant changes. | YES  (5-6%) | FiO_2_ (0.14) (NH) | Incremental treadmill test until exhaustion:  At 6-7.5 km/h at 2%; steps of 3 minutes with increased 2% of ramp grade. |
| Salazar-Martinez et al.(Salazar-Martínez, Gatterer, Burtscher, Orellana, & Santalla, 2017) | 16 physically active subjects. n=9 men, n=7 women.  Powerbreathe:  RMT group (n=8) 2x30 resp/d at 50% MIP.  Control group (n=8) no RMT.  6 weeks | No differences between genders.  PPO increased significantly only in RMT group in normoxia post training (5%) and (2%) in hypoxia (p<0.05).  Despite reducing VO_2max_ (5%) in hypoxia, RMT increased cycling performance (+7.3%).  After the experimental period, WTT_mean_ (W)  and WTT_mean_ (W/Kg) were significantly higher in normoxia and hypoxia only in the RMT group (p < 0.05).  RMT group improved TT performance in normoxia (10%) and hypoxia (6%). | RMT improved VE/VCO_2_ slope (−7.95%) in hypoxia.  ↑ MIP (28%) with RMT (no improvement in the control group) (p<0.05).  Correlations between MIP and and TT performance in normoxia (r=0.69) and in hypoxia (r=0.67) | N/A | N/A | FiO_2_ (0.16) (NH) | Cycle ergometer:  normoxic and hypoxic incremental tests: 4 minutes warm-up at 50 watts; 25 watts increase per min.  TT 90 min later: 10' all-out. |
| Esposito et al. (6) | 9 physically active subjects.  Isocapnic hyperpnea.  RMT group: 10-20’ 5d/week.  No control group.  8 weeks | No improvements in sub-maximal cycling performance (hypoxia and normoxia) (W_max_).  RMT did not affect maximum HR or VO_2max_ neither in hypoxia nor normoxia. | Post-RMT, FVC (+8%, P < 0.05), FEV1 (+9%,  P < 0.05), PEF (+8%, P < 0.05), VC (+7%, P < 0.05), ERV (+8%, P < 0.05) were significantly increased. On the contrary, RV (−20%, P < 0.01) and RAW (−17%, P < 0.01) were significantly decreased.  RMT increased expired minute volume (+12%) and alveolar ventilation in hypoxia (+13%).  RMT increased pulmonary function (static and dynamic volumes) and alveolar-arterial gradient in hypoxia.  MIP increased (+75%).  No changes in the control group. | N/A | NO | FiO_2_ 0.11  normobaria | Cycle ergometer:  Incremental tests in normoxia and hypoxia 5 bouts of 5 minutes with increasing intensity separated by 5 minutes. |
